# Capturing a rising star: the emerging role of astrocytes in neural circuit wiring and plasticity–lessons from the visual system

**DOI:** 10.1117/1.NPh.10.4.044408

**Published:** 2023-09-26

**Authors:** David Foubert, Finnley Cookson, Edward S. Ruthazer

**Affiliations:** Montreal Neurological Institute-Hospital, Integrated Program in Neuroscience, McGill University, Montreal, Quebec, Canada

**Keywords:** calcium imaging, neuron-glia interactions, astrocytes, visual system, circuit wiring, development, neuromodulation

## Abstract

The increasingly widespread use of calcium imaging to explore the nature of neuronal activity and circuits has unexpectedly revealed the ubiquitous presence and significance of astrocytic activity. Here, we present a brief review of visual system development, placing it in the context of recently identified roles of astrocytes in the modulation of neuronal responses and circuit plasticity, through their responses to sensory stimuli and the release of gliotransmitters.

The orderly representation of sensory space in the form of retinotopic maps in the visual system makes it an accessible and intuitive model system in which to investigate the molecular and experience-dependent mechanisms responsible for establishing precise circuit wiring in the brain. Increasingly, it has become clear that glial cells, long-neglected in early studies, are in fact fundamental partners in this process. Our well-established understanding of the key mechanisms that underlie visual system development therefore offers a useful starting point for investigating the involvement of glia in circuit refinement and function.

The initial pathfinding of retinal ganglion cell (RGC) axons from the eye relies on a diverse set of molecular guidance cues, including slits, netrins, semaphorins, and ephrins to ensure projecting neurons reach their targets and synapse with their proper partners.^1^^,^^2^ Subsequent retinotopic projection refinement exploits patterned neural activity, which in mammals takes the form of spontaneous retinal waves prior to eye-opening.^3^ The importance of retinal waves in refining RGC axon arbor termination zones in the superior colliculus is underscored by studies in ß2-nicotinic acetylcholine receptor knockout mice, which lack retinal waves. RGC projections in these mice fail to fully refine, occupying a broader area with less dense local arborization.^4^ Moreover, retinal waves have been found to propagate throughout the visual system where they may provide further wiring guidance in higher visual areas during critical periods for topographic specification.^5^^,^^6^

By contrast, RGCs in most externally fertilized species, including common experimental models such as zebrafish (*Danio rerio*) and African claw-toed frogs (*Xenopus laevis)*, respond to visual input starting from much earlier developmental timepoints, obviating the need for spontaneous retinal waves to provide patterned activity.^7^ The developing retinotectal projection in these animals is primed to undergo experience-expectant refinement driven by optic flow-generated retinal activity.^8^ This property of early visual responsiveness in the retinotectal systems of small, transparent aquatic organisms makes them particularly well-suited for studies of circuit refinement in early development, and much work to date has focused on structural remodeling of single RGC axon arbors or tectal dendritic fields, reviewed in Kutsarova et al.^9^ The translucency and small size of larval zebrafish and Xenopus tadpoles also account for their having been adopted as subjects in some of the earliest intravital imaging studies of the vertebrate brain, ranging from *in vivo* time-lapse imaging of single neuron morphology to whole-brain calcium imaging.

## 1 Calcium Imaging Reveals Sensory Maps in Both Neurons and Astrocytes

**Fig. 1:**
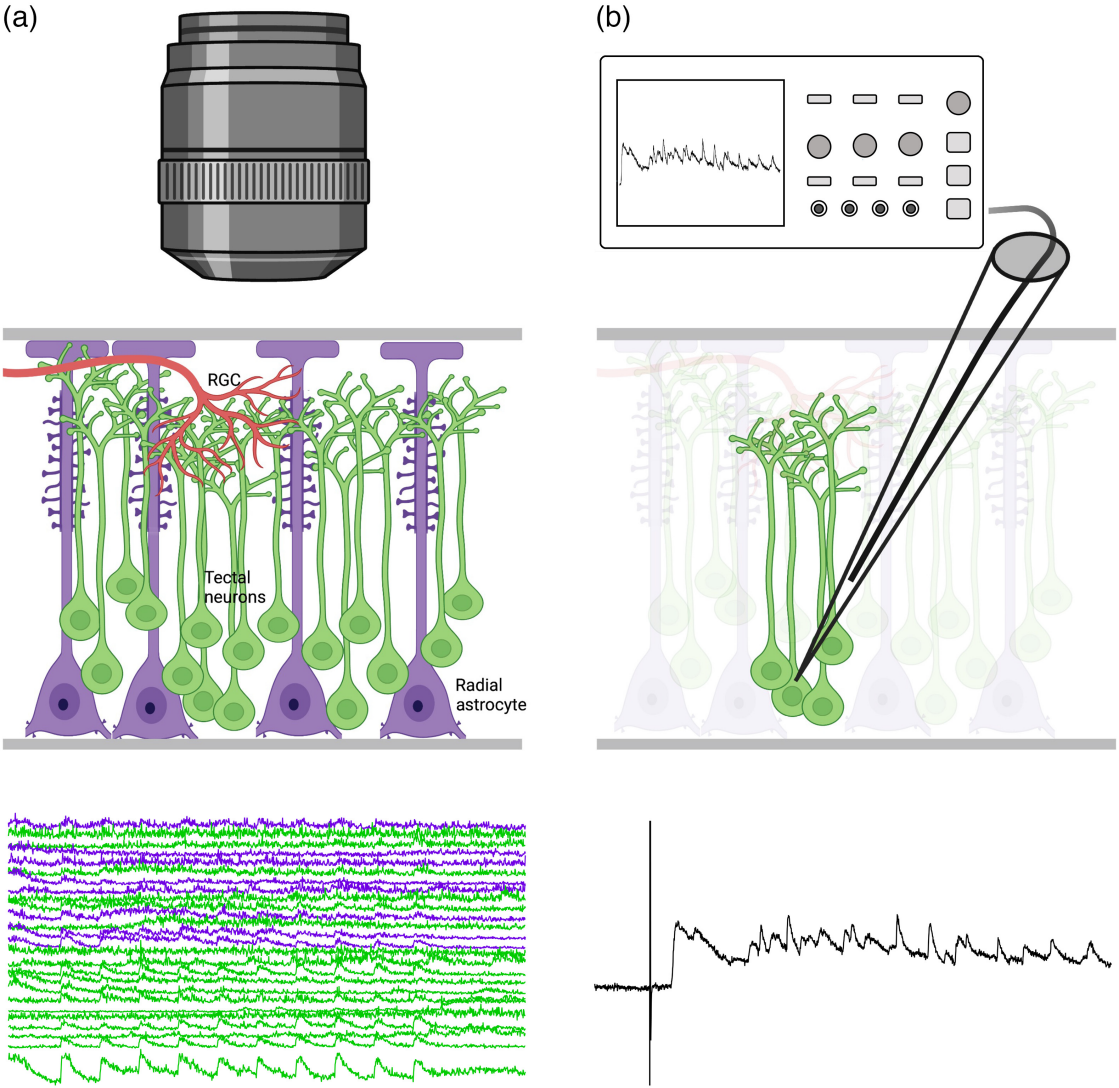
Comparing the application of (a) optical and (b) electrophysiological methods to the *X. laevis* optic tectum. Electrophysiological methods, although superior in temporal resolution and in detecting spiking, cannot achieve the subcellular spatial resolution, cell-type specificity, and spatial coverage provided by calcium imaging (images generated using BioRender).

Until recently, it was unclear whether the initial functional organization of RGC inputs, especially to the immature Xenopus tadpole optic tectum, was sufficiently organized to manifest as orderly retinotopic maps. Initial extracellular electrophysiological tectal mapping studies in Xenopus tadpoles indicated remarkably poor retinotopic organization at these early developmental stages.^10^ While electrophysiological methods have the benefit of reporting cellular responses at high temporal resolution, they are poorly suited for mapping neural connectivity at fine sub-cellular spatial resolution in the small brains of developing organisms (Fig. 1). Interestingly, more recent investigation utilizing *in vivo* calcium imaging has indeed revealed an impressive degree of fine-scale topographic order at subcellular resolution within the three-dimensional structure of the Xenopus tectal neuropil and a role for neuronal activity in further refining these retinotopic maps.^11^

In addition to improved spatial resolution, the possibility of imaging calcium dynamics over large volumes of brain tissue has also heralded a shift from traditional electrophysiological recordings from single or small numbers of neurons, to observing network-wide response properties.^12^ The use of chemical fluorescent calcium indicators first revealed that astrocytes, far from being passive support cells, are themselves capable of exhibiting robust responses to external stimuli in the form of calcium transients.^13^ Genetically encoded calcium indicators (GECIs) have largely replaced their chemical predecessors due to the ability to obtain broad expression in defined cell types with minimal invasiveness.^14^

One surprising set of findings to emerge from *in vivo* calcium imaging is the degree to which astrocytes can exhibit sensory-evoked calcium transients that are highly selective for stimulus features, and reflect a degree of functional selectivity rivaling that of neurons. In ferret primary visual cortex, astrocytes respond to visual stimuli with defined receptive fields and sharp tuning to stimulus features such as orientation preference.^15^ These responses, which are detected primarily in the astrocytic somata, are delayed by up to several seconds relative to their neighboring neurons.^16^ Similarly, in the Xenopus optic tectum, radial astrocytes have been shown to respond to visual loom stimuli with a latency of 2 to 3 s later than nearby neurons.^17^ Interestingly, even after full pharmacological blockade of all glutamatergic receptors on tectal neurons, these glial responses persist in full, suggesting that they are driven, not by signals released by local tectal neurons, but directly by RGC axon glutamate release. This was confirmed by live calcium imaging in tadpoles expressing the GECI GCaMP6s, in which the visually evoked responses of radial astrocytes were abolished by blockade either of excitatory amino acid transporters (EAATs) expressed in astrocytes or of the sodium–calcium exchanger that pumps calcium into radial astrocytes while exchanging the sodium that EAATs import into the cell together with glutamate for calcium but not by blocking glutamate receptors on postsynaptic tectal neurons. This finding demonstrates a critical role for the sodium–calcium exchanger in mediating direct signaling by glutamate from RGC axon terminals to drive sensory-driven ${\mathrm{Ca}}^{2+}$ events in glia during early development.

In addition to sensory-driven calcium transients, astrocytes in the brains of mice, as well as fish and frogs, can respond robustly to neuromodulatory influences, such as norepinephrine (NE) released during locomotion.^18^ Inhibition of alpha1-adrenergic receptors significantly reduces neuromodulator-driven global activity in primary visual cortex, unmasking the smaller visually-driven responses in microdomains.^16^ As opposed to somatic calcium elevations, astrocyte microdomains are mostly found locally within fine peripheral processes near neuronal synapses and can respond to single synaptic events with rapid local calcium elevations on a timescale more similar to neuronal events.^19^^,^^20^ Using a clever anterograde viral transduction method to target expression of a GECI to astrocytes that interact with thalamocortical axons in the whisker cortex in mouse, Georgiou et al.^21^ showed that sensory-evoked calcium microdomains in astrocytes could be observed as hotspots where calcium elevation was strongly elevated during volitional locomotor behavior, presumably via NE release, and that the locations and properties of these behaviorally modulated hotspots were stable over days. Taken together, these findings suggest that astrocytes do not only read out local glutamate release but also modulate their responses to these stimuli depending on behavioral state and neuromodulation. How might such differences in glial activation, based on behavioral state and neuromodulatory input, impact the mechanisms that underlie developmental circuit refinement in the retinotectal system?

## 2 Gliotransmission Mediates Metaplasticity

In the developing retinotectal (retinocollicular) system, N-methyl-D-aspartate receptor (NMDAR) blockade prevents normal circuit refinement, measured both functionally and anatomically.^11^^,^^22^^–^^26^ A useful set of experimental models for studying NMDAR-dependent mechanisms in Xenopus development are retinotectal spike-timing-dependent long-term potentiation and depression.^27^ Spike-timing-dependent plasticity has been shown to induce receptive field shifts, suggesting its relevance for circuit refinement.^28^ Here too, optical approaches have proven useful for studying network-scale plasticity. Using calcium imaging to quantify in parallel the response changes of dozens of tectal neurons while imaging single-cell morphological remodeling, it was shown that plasticity-inducing stimuli that strengthen functional responses also promote dendritic growth and stabilization; conversely, stimuli that depress tectal responsiveness lead to dendritic branch loss.^29^^,^^30^ Similarly, on the presynaptic side, RGC axons that fire synchronously with neighboring inputs engage an NMDAR-dependent Hebbian branch stabilization mechanism, whereas the axons that fire out of synchrony with other inputs exhibit destabilization and exploratory branch induction, referred to as Stentian plasticity.^9^^,^^31^

Astrocytes in many brain areas are able to modulate synaptic efficacy through their calcium transient-driven release of gliotransmitters, including D-serine, adenosine triphosphate (ATP), and glutamate. D-serine is a co-agonist of the NMDAR that greatly enhances calcium currents through NMDARs and thus has been observed to act as a metaplasticity factor to favor LTP over LTD.^32^ Electrophysiological experiments have shown that calcium-mediated D-serine release from astrocytes promotes NMDAR-dependent LTP in rodent cornu Ammonis 1.^33^^–^^35^ In the retinotectal system, D-serine has been shown to promote synaptic maturation through the addition of aminomethylphosphonic acid type glutamate receptor, and Hebbian axonal stabilization through its enhancement of NMDAR function.^36^

Gliotransmitters ATP and glutamate have also been linked to changes in synaptic efficacy and thus may contribute to the plasticity of the retinotectal system. ATP can either act directly on purinergic receptors or can be degraded to adenosine to act on a distinct family of G-protein-coupled receptors. Although not yet shown in the optic tectum, activation of astrocytes by NE was shown to trigger ATP release and activation of P2X(7) receptors on neurons to mediate an increase in postsynaptic efficacy in rat hypothalamus.^37^ Furthermore, glutamate itself is believed to act as a gliotransmitter in some circumstances. For example, it has also been demonstrated that glutamate-induced glutamate release from Bergmann glia in the cerebellum amplifies excitatory signals by contributing to synaptically mediated currents.^38^

## 3 Modulation of Brain States through Astrocyte Signaling

Given the large, tiled domains serviced by the fine processes of astrocytes, bringing each in contact with about 100,000 synapses, as well as their potential to communicate with one another via gap junctions, they are well-positioned to regulate circuit-wide neural activity and brain states. It therefore makes sense that neuromodulatory inputs might leverage the intimate access that astrocytes have with synapses across a brain region to both expand and better target their influence on the network.

In mice, stimulation of nucleus basalis, whose activity normally correlates with elevated attentional states, drives modulatory acetylcholine release that activates muscarinic receptors on astrocytes in primary visual cortex.^39^ This, in turn, causes slow currents in cortical neurons that enhance NMDAR-mediated signaling, an outcome that is prevented when calcium transients in the astrocytes are blocked. Impressively, the pairing of cholinergic stimulation with presentation of visual stimuli at a given orientation specifically enhanced the response of cortical neurons to the paired orientation.

Whole-brain calcium imaging in actively swimming zebrafish revealed that radial astrocytes accumulate evidence of swimming futility from noradrenergic inputs, which fire when motor activity is experimentally mismatched with visual feedback.^40^ The radial astrocytes exhibit bursts of intense pan-network calcium transients that appear to prompt gamma-aminobutyric acid neurons to arrest motor output in the hindbrain.

Astrocyte activity also appears to precede transitions between slow wave sleep and wakefulness in the mammalian brain. Disruption of calcium signaling in astrocytes impairs slow wave sleep and results in sleep pattern anomalies.^41^ Astrocytic calcium signaling in cortex is strongly correlated with changes in arousal state. Arousal-associated NE release precedes astrocyte calcium activity, and the blockade of alpha1a-adrenergic (Adra1a) receptors reduces the correlation between astrocytes and cortical state.^42^ Conditional knockout of Adra1a in astrocytes increased arousal-related neuronal activity and asynchronous neuronal activity. In contrast, activation of glial Adra1a receptors increased cortical synchrony, suggesting that NE-driven astrocyte signaling, in contrast to its direct effects on neurons, mediates the transition back from high to low arousal states.

Recent work from our lab indicates that NE is similarly effective in driving radial astrocyte activation in the optic tectum of developing Xenopus tadpoles and that much like mammalian cortex this glial activity profoundly alters neuronal excitability and selectivity for visual stimuli.^43^ Moving forward, small transparent models such as the Xenopus visual system can offer a powerful platform in which to combine opto- or chemogenetic control of glial activity with classic neurophotonic assays for structural and functional plasticity of the developing retinotectal system. The traditional view of the experience-dependent fine-tuning of developing brain circuits as an exclusively neuronal phenomenon is due to undergo significant modulation in coming years.
